# A Modified Fatigue Life Prediction Model for Cyclic Hardening/Softening Steel

**DOI:** 10.3390/ma18143274

**Published:** 2025-07-11

**Authors:** Zhibin Shen, Zhihui Cai, Hong Wang, Bo Xu, Linye Zhang, Yuxuan Song, Zengliang Gao

**Affiliations:** 1 Wenzhou Special Equipment Inspection & Science Research Institute, Wenzhou 325000, China; zhibinshen@wzsei.com (Z.S.); zhihui.cai@wzsei.com (Z.C.); 2 Huzhou Special Equipment Inspection Center, Huzhou 313099, China; wh@hztjy.com; 3 Ningbo Special Equipment Inspection and Research Institute, Ningbo 315048, China; xb@nbtjy.com; 4 Institute of Process Equipment and Control Engineering, College of Mechanical Engineering, Zhejiang University of Technology, Hangzhou 310023, China; songyux@zjut.edu.cn (Y.S.); zlgao@zjut.edu.cn (Z.G.); 5 Institute of Innovation Research of Shengzhou and Zhejiang University of Technology, Shengzhou 312400, China

**Keywords:** hysteresis loop, cyclic plastic strain, flow stress, fatigue life

## Abstract

The accumulation of fatigue damage is primarily caused by cyclic plastic deformation. In low-cycle fatigue, cyclic plastic deformation is the dominant deformation mode. In high-cycle fatigue, although most deformation is elastic, plastic deformation may still occur in localized regions of stress concentration and plays a critical role in the initiation of fatigue cracks. Considering that cyclic plastic deformation can be characterized by hysteresis loops, this study modifies the flow stress equation and the cyclic plastic deformation relationship based on stress–strain hysteresis loops at half-life cycles under different strain amplitudes. An improved model for life prediction that incorporates the effects of strain amplitude is proposed. The results of experiments on 310S stainless steel and 1045 carbon steel demonstrate that the model achieved prediction errors within a factor of two and provided reliable predictions for both high-cycle and low-cycle fatigue life across the entire ε-N curve.

## 1. Introduction

Metals often endure complex cyclic loading during service, making them prone to fatigue failure. This poses significant challenges to structural safety in fields such as aerospace, nuclear power, automotive, and marine engineering. In recent years, scholars have conducted systematic studies on the fatigue performance of various metallic materials based on theoretical and experimental approaches [1,2]. The main methods for predicting fatigue life currently include the stress–life curve (S-N curve)-based method [3,4], the strain–life curve (ε-N curve)-based method [5,6,7], the fracture mechanics method [8,9,10], the energy dissipation-based method [11,12], the nano-indentation method based on material performance parameters [13,14], numerical simulation, and finite element analysis [15,16]. However, these methods have certain limitations. For example, they cannot simultaneously account for both high-cycle and low-cycle fatigue behavior; they are strongly influenced by the material’s microstructure or grain size; and they are often restricted to specific loading conditions or material states.

Since materials undergo irreversible plastic deformation and accumulate micro-damage under external loading, it is reasonable to use plastic strain as a fatigue damage parameter [17]. The Coffin–Manson formula is a classic prediction method based on the ε-N curve and plastic strain. It can accurately describe the accumulation of plastic strain damage in low-cycle fatigue. Xu et al. [18] proposed a flow stress-based damage model that provides a good fit for the ε-N curve in low-cycle fatigue, with a relative error within 10%. Liu et al. [19] proposed a life-prediction model based on the modified plastic strain energy according to the evolution law of plastic strain energy and successfully predicted the fatigue life of 45CrNiMoVA steel. The modified model made the error distribution more concentrated, reducing the prediction error from 13.73% to 7.68%. Hu et al. [20] established an energy dissipation-based model based on the plastic strain energy dissipation process to predict low-cycle fatigue life in the candidate AM alloy. The proposed model achieved a fatigue-life prediction accuracy that reduced the error band to 1.5×, outperforming traditional models such as the Manson–Coffin model, the plastic strain energy model, and the total plastic strain energy model. Prediction models based on plastic strain can usually accurately predict low-cycle fatigue life, but their effectiveness in high-cycle fatigue is less satisfactory. This is because in low-cycle fatigue, cyclic plastic deformation is the predominant deformation mode, and the fatigue life is primarily determined by the plastic strain amplitude $\varepsilon^{p}$. For high-cycle fatigue, although most deformation is elastic, plastic deformation may still occur in localized areas of concentrated stress and plays a critical role in initiating fatigue cracks [21,22]. At the microscopic level, cyclic plastic deformation accelerates fatigue damage through mechanisms such as dislocation accumulation, formation of slip bands, and crack initiation. At the macroscopic level, it influences fatigue life by altering the stress–strain response, such as cyclic hardening or softening. Understanding the relationship between cyclic plastic deformation and fatigue damage and accurately predicting the strain fatigue life of metallic materials are crucial for optimizing material design and enhancing fatigue resistance.

In strain fatigue testing, phase transformation directly affects the fatigue behavior of materials, including their stress–strain response and cyclic hardening/softening behavior [23,24]. In addition, phase transformation may also lead to uneven material structure [25], affecting local stress concentration and the initiation paths of fatigue cracks [26]. This places higher demands on the accuracy of fatigue life prediction models [27]. Many parameters in fatigue life models are determined based on the initial microstructure. If a phase transformation occurs during the fatigue loading, these parameters no longer reflect the current microstructural state. As a result, the model may underestimate or overestimate the fatigue life, leading to a larger deviation between the actual and predicted lifespans. If Martensitic phase transformation is unavoidable, it is generally necessary to develop prediction models that can dynamically account for the effects of phase transformation. Therefore, for our experiments, we selected 1045 carbon steel and 310S stainless steel, which do not undergo phase transformation during strain fatigue tests. These materials also exhibit different cyclic deformation behaviors, enabling further verification of the accuracy of the life prediction model based on plastic strain for various materials. In this study, the strain–fatigue life prediction for 1045 carbon steel and 310S stainless steel was conducted using a modified model based on plastic strain and flow stress. This model can effectively predict both high cycle and low cycle fatigue life across the entire ε-N curve by correcting the equation between plastic strain and flow stress according to the strain amplitude.

## 2. Materials and Methods

### 2.1. Materials and Experiments Methods

**Figure 1:**
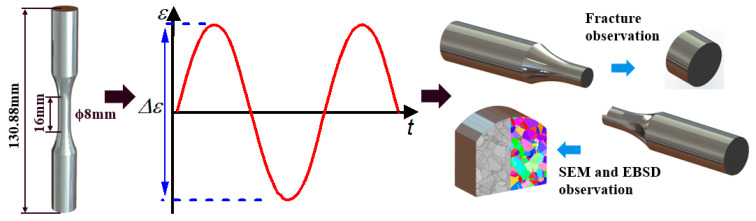
The experimental process included specimen size; strain fatigue test; fracture surface, SEM, and EBSD observation.

Hot-rolled 1045 carbon steel and cold-rolled 310S Austenitic stainless steel were used in this work. The chemical compositions detected via energy-dispersive spectrometry (EDS) are shown in Table 1. Fatigue specimens were machined from commercially acquired bars. As shown in Figure 1, the specimens had a diameter of 8.0 mm and a gauge length of 16 mm. To minimize the initial damage caused by specimen machining, the following parameters were used during finish turning: a feed depth of 0.02 mm, ten passes, and a feed rate of less than 0.15 mm/r for each pass.

**Figure 2:**
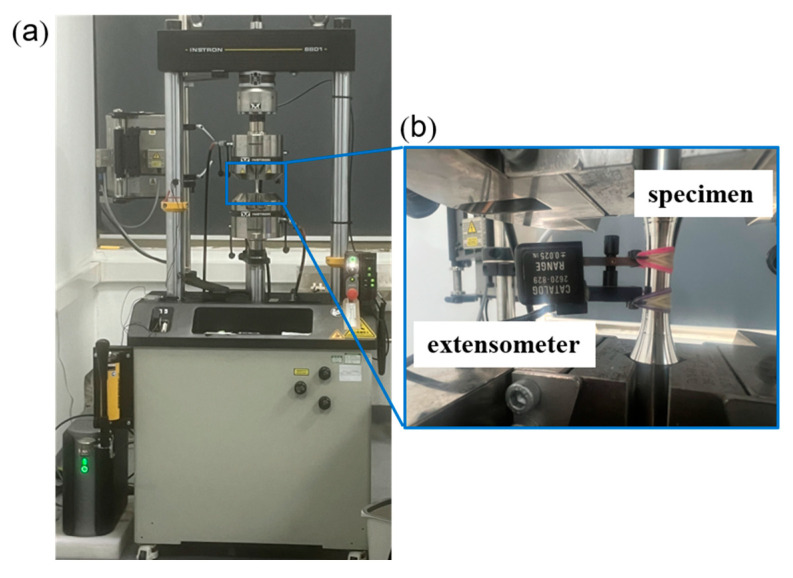
(**a**) Fatigue testing device and (**b**) extensometer and specimen during fatigue test.

Fatigue experiments were conducted using a servo-hydraulic tension-compression testing machine (Instron 8801, High Wycombe, UK, shown in Figure 2) in ambient air. Fully reversed strain-controlled constant-amplitude fatigue experiments were carried out using 1045 steel specimens with strain amplitudes ranging from 0.165% to 1.0% and 310S Austenitic stainless specimens with strain amplitudes ranging from 0.25% to 1.0% with a sinusoidal wave form (shown in Figure 1). The loading frequency ranged from 0.2 Hz to 5.0 Hz dependent on the strain amplitude. In the fatigue tests, the strain was measured using an extensometer with a gauge length of 12.7 mm and a strain range of ± 5%. The stress–strain response of selected loading cycles was considered and at least 200 data points were recorded for each of the selected cycles.

Scanning electron microscopy (SEM) and electron back-scattered diffraction (EBSD) were used to characterize the microstructures of the material before and after the fatigue experiments. As shown in Figure 1, for these specimens after fatigue failure, the EBSD samples were cut within the gauge section of fatigue specimens, away from the macroscopic fatigue cracks, and the samples were mechanically polished to a mirror-like surface. The fracture morphology of the fatigued specimens was observed using SEM to determine the sites of crack initiation and early crack propagation.

**Figure 3:**
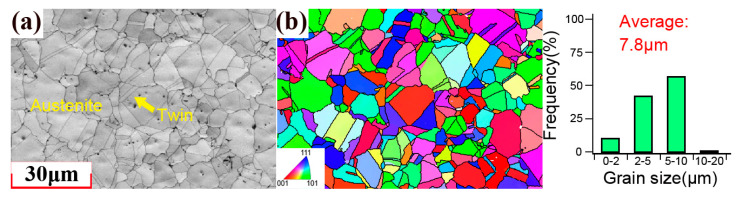
Cross-sectional EBSD image of the 310S specimen.

**Figure 4:**
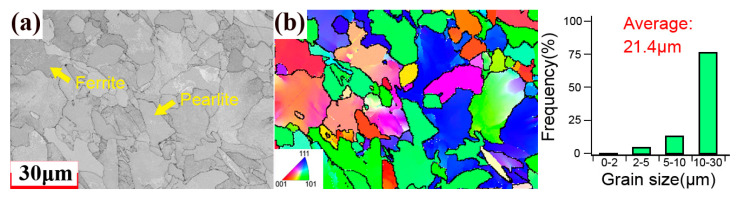
Cross-sectional EBSD image of the 1045 specimen.

Figure 3 shows the microstructure of the 310S stainless steel obtained using EBSD. The microstructure was composed of equiaxed austenite crystals and the average grain size was approximately 7.8 μm. Twins can be observed inside the grains. Figure 4 shows the microstructure of the 1045 carbon steel obtained using EBSD. The microstructure was composed of ferrite and pearlite, and the average grain size was approximately 21.4 μm. Figure S2 shows the KAM map of the 1045 and 310S specimens before the fatigue test. The results indicated that the initial KAM inside the specimen was very low, which suggests a low initial dislocation density. The XRD results indicated that the residual stress on the surface of the specimen before the experiment was almost zero. Therefore, the effects of residual stress and initial damage are not considered in the subsequent analysis.

### 2.2. Fatigue Life Estimation Method

**Figure 5:**
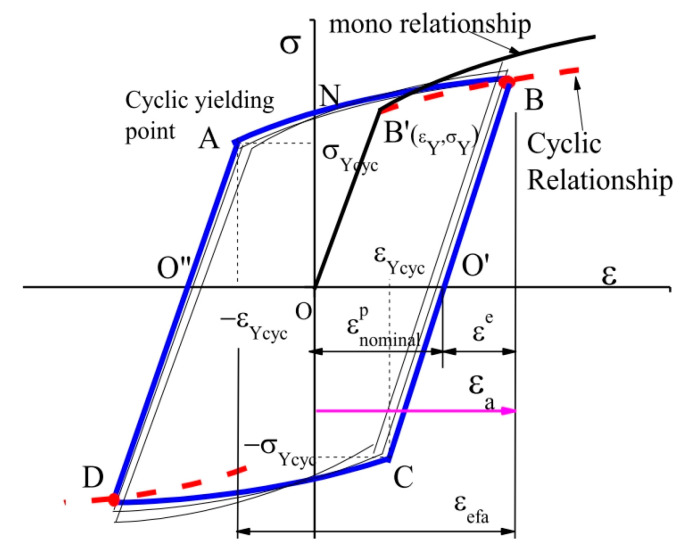
An ideal saturated hysteresis loop [18].

Xu [18] considered the ideal saturated hysteresis loop under fully reversed strain-controlled low-cycle fatigue, as shown in Figure 5 (ABCD). Cyclic yielding happens at Point A and C in the saturated cycle, cyclic plastic deformation appears along the path A→B and C→D. The cyclic yield strength $\sigma_{Ycyc}$ and cyclic yield strain $\varepsilon_{Ycyc}$ can be regarded as the cyclic yield properties of the material, which differ significantly from the original yield strength and strain obtained through uniaxial tension. The cyclic yield strain is also different from the commonly defined plastic strain amplitude $\varepsilon_{anornimal}^{p}$ of the hysteresis loop.

Plastic deformation progresses along the path A→B and C→D. At point A, the cyclic plastic strain is 0, and it is $\varepsilon_{Ycyc}+\varepsilon_{a}$ at point B. Taking the half cycle as an example, the cyclic stress of the hysteresis loop along the path A→B can be expressed in terms of cyclic yield strength $\sigma_{Ycyc}$ and cyclic yield strain $\varepsilon_{Ycyc}$ as follows:

(1) $$\sigma_{a}\left(\varepsilon_{a}\right)=\sigma_{Ycyc}+h{\left(\varepsilon_{a}+\varepsilon_{Ycyc}\right)}^{n}\;\mathrm{along}\;A\to B\;$$

where *h* and *n* denoted hardening effects of cyclic plastic deformation path A→B. Since fatigue damage accumulation is mainly caused by progressive cyclic plastic deformation, cyclic plastic strain and cyclic stress can be used to characterize the fatigue behavior of materials. The cyclic stress along path A→B is also referred to as the flow stress by Xu. When the strain amplitude reaches its maximum value, the stress amplitude in Equation (1) corresponds to the maximum stress $\sigma_{a(max)}$ in the hysteresis loop.

It is worth noting that the cyclic stress–strain relationship can also be described using the maximum stress–strain points of the hysteresis loop under different strain amplitudes:

(2) $$\sigma_{a}(\max)=\sigma_{Y}+h_{a}{\left(\varepsilon_{a}(\max)-\varepsilon_{Y}\right)}^{n_{a}}$$

where $\varepsilon_{Y}$ is the original yield strain, and *h_a_* and *n_a_* denote hardening effects of the cyclic stress–strain curve. According to Equations (1) and (2), the cyclic yield stress and strain can be described by the following equation:

(3) $$\sigma_{Ycyc}=\sigma_{Y}+h_{a}{\left(\varepsilon_{a}-\varepsilon_{Y}\right)}^{n_{a}}-h{\left(\varepsilon_{a}+\varepsilon_{Ycyc}\right)}^{n}$$

Cyclic yielding strength and strain are both dependent on applied strain amplitude. The cyclic yield strain in Equation (3) can also be simply fitted using the applied strain amplitude:

(4) $$\varepsilon_{Ycyc}=k\varepsilon_{a}+b$$

where *k* and *b* can be regarded as material constants.

**Figure 6:**
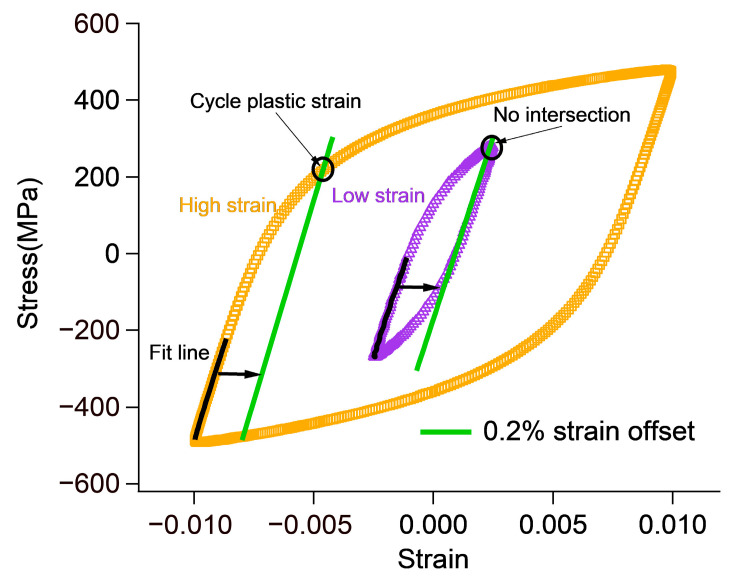
Hysteresis loop and 0.2% offset line at high and low strain amplitudes.

In practical experiments, the hysteresis loop of the materials is typically a smooth curve without a distinct cyclic yield point. To determine the cyclic yield strength and strain, Xu introduced a 0.2% residual strain offset line as shown in Figure 6. Research by Xu and Roy et al. has shown that using linear methods to determine approximate cyclic yield strength can also achieve excellent life-prediction results. Under low-cycle fatigue with high strain amplitude, the cyclic yield point can be determined by the intersection of the offset line and the hysteresis loop. However, at lower strain amplitudes, the 0.2% offset line does not intersect the hysteresis loop, making it impossible to obtain the cyclic yield stress and strain in this way. In fact, cyclic plasticity still exists under low strain amplitudes, and it is unreasonable to exclude the effect of plastic strain $\varepsilon^{p}$ using the 0.2% offset line proposed by Xu. In this paper, $\varepsilon^{p}$ obtained using the 0.2% offset line is compared with the classical $\varepsilon_{anornimal}^{p}$, and the larger value of the two is taken. Therefore, at lower strain amplitudes, the classical $\varepsilon_{anornimal}^{p}$ is used to define the cyclic yield point. As a result, there are two sets of cyclic yield stress–strain data.

Considering the accumulation of fatigue damage caused by cyclic plastic deformation. Xu established the following damage model for flow stress:

(5a) $$\frac{dD}{dt}=c\frac{1}{1-D}\frac{d\sigma \left(t\right)}{dt}{\left(\frac{\sigma \left(t\right)}{1-D}\right)}^{\beta }H\left(\sigma \left(t\right)\right)$$

(5b) $$H=\left\{\begin{array}{ll} 1-{\left(\frac{\left(1-D\right)\sigma_{fY}}{\sigma \left(t\right)}\right)}^{\gamma } & \;\mathrm{when}\;\frac{\sigma \left(t\right)}{1-D}>\sigma_{fY}\\ 0 & \;\mathrm{when}\;\frac{\sigma \left(t\right)}{1-D}<\sigma_{fY} \end{array}\right.$$

where σ(t)/(1−D) is the effective stress, taking the damage effect into account, *β* and *γ* are life and curve factors, respectively, *c* is a proportional coefficient of damage accumulation, *H* is a curve function introduced to describe curve features, $\sigma_{fy}$ is the fatigue limit. The formula for predicting strain fatigue life based on flow stress can be expressed as:

(6a) $$\sigma_{a}^{\beta +1}N_{f}={\left[\sigma_{Ycyc}+h{\left(\varepsilon_{a}+\varepsilon_{Ycyc}\right)}^{n}\right]}^{\beta +1}N_{f}=CI\left(\varepsilon_{a}\right)$$

(6b) $$I\left(\varepsilon_{a}\right)=\int_{D_{0}}^{D_{c}}\frac{{\left(1-D\right)}^{\beta +1}}{\left[1-\frac{\beta +1}{\beta -\gamma +1}{\left(\frac{\left(1-D\right)\sigma_{fY}}{\sigma_{a}}\right)}^{\gamma }\right]-{\left(\frac{\sigma_{0}}{\sigma_{a.}}\right)}^{\beta +1}\left[1-\frac{\beta +1}{\beta -\gamma +1}{\left(\frac{\left(1-D\right)\sigma_{fY}}{\sigma_{0}}\right)}^{\gamma }\right]}dD$$

where:

(7a) $$\sigma_{0}=\left\{\begin{array}{ll} \sigma_{Ycyc} & \;\mathrm{if}\sigma_{fY}\leq \sigma_{Ycyc}\\ \left(1-D\right)\sigma_{fY} & \;\mathrm{if}\sigma_{fY}>\sigma_{Ycyc} \end{array}\right.$$

(7b) $$\sigma_{a}=\sigma_{Ycyc}+h{\left(\varepsilon_{a}+\varepsilon_{Ycyc}\right)}^{n}$$

where *C* is the life coefficient, *D*_0_ is the initial damage which can be set as 0 for non-used metals, and *D_c_* is the critical damage. According to Chaboche’s theory, *D_c_* can be determined by instantaneous fracture condition as follows:

(8) $$\;D_{c}=1-\frac{\sigma_{a}}{\sigma_{b}}$$

where $\sigma_{b}$ is the ultimate tensile strength of the material.

## 3. Fatigue Test Results

### 3.1. Fatigue Behavior

**Figure 7:**
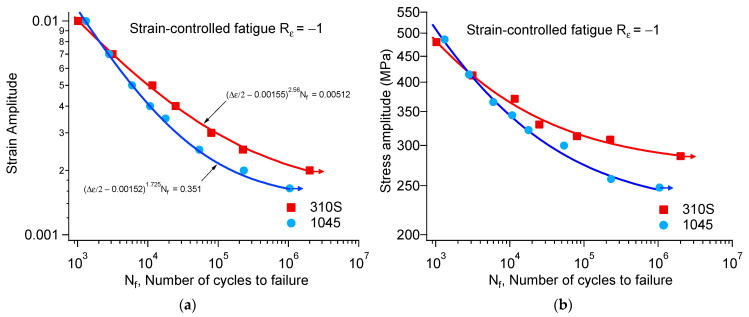
(**a**) Strain–life fatigue curves and (**b**) stress–life fatigue curves for 310S and 1045 steels.

Fatigue results are summarized in Table 2 and Table 3 for 310S and 1045 specimens, respectively. The strain–life fatigue curves and stress–life fatigue curves of 310S and 1045 steel are shown in Figure 7a and Figure 7b, respectively. The following three-parameter equation is usually used to fit the experimental data of strain-controlled fatigue. For most metallic materials, the experimental data can be reasonably fitted by one set of parameters of Equation (9). The solid lines in the figure have been added to display the best fit results. The arrow after a marker indicates a run-out test.

(9) $${\left(\frac{\Delta \varepsilon }{2}-\varepsilon_{0}\right)}^{\xi }N_{f}=C$$

where Δ*ε*/2 is the strain amplitude, *N_f_* is the number of cycles to failure, and *ε*_0_, ξ, and C are the constants determined by fitting the experimental data.

It is worth noting that the loading frequency ranged from 0.2 to 5.0 Hz, depending on the strain amplitude. The main purpose was to complete the fatigue tests without causing the specimens to overheat. Although the frequency varied by an order of magnitude, the strain rate was still maintained within the range of 0.001 to 0.01. Luo pointed out that when the strain rate does not exceed 0.012, it has little effect on the strain fatigue behavior of steel. Therefore, the different frequencies used in this paper did not significantly affect the fatigue test results.

Under cyclic loading, fatigue strength is the stress amplitude corresponding to a given fatigue life. As a general practice, the stress–life curve, where the stress amplitude is plotted versus the fatigue life, is used for the assessment of the fatigue strength. The stress amplitude at approximately half of the fatigue life from strain-controlled fatigue experiments is used to obtain the stress-fatigue life curve. To facilitate discussion, this study used the stress amplitude corresponding to a fatigue life of 10^6^ cycles as the fatigue limit. For convenience of observation, the stress amplitude–life curves were also fitted. Since the stress amplitude data were obtained through strain fatigue, the same three-parameter equation was also used for fitting, but the strain amplitude in the equation was replaced with the stress amplitude in Equation (1). The stress–life fatigue curves of 1045 and 310S steel are shown in Figure 7b.

**Figure 8:**
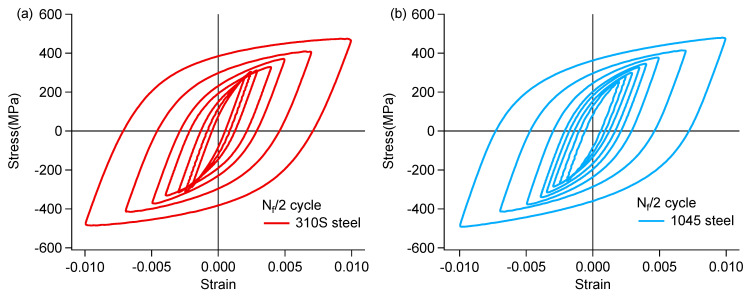
Stress–strain hysteresis loops at half fatigue lives of the (**a**) 310S steel and (**b**) 1045 steel.

**Figure 9:**
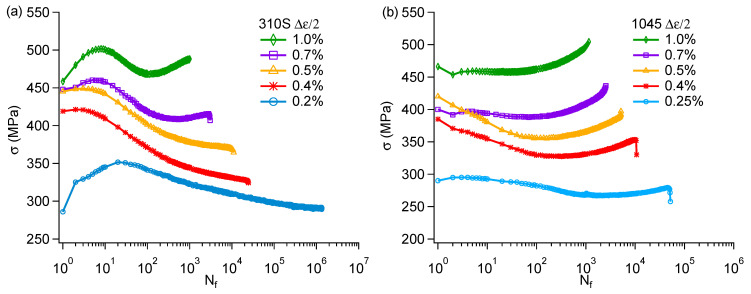
Stress response under different strain amplitudes: (**a**) 310S steel and (**b**) 1045 steel.

Stress–strain hysteresis loops not only reflect the macroscopic mechanical behavior of materials but also reveal their microscopic damage mechanisms and fatigue characteristics. By analyzing the shape, area, and evolution patterns of hysteresis loops, fatigue life can be predicted and material properties evaluated. The hysteresis loop at half-life typically represents the cyclic stress–strain behavior of a material during stable fatigue cycling and is therefore commonly used as a reference for prediction of fatigue life [19,20]. Figure S3 shows the hysteresis loops of 310S specimens at different cycle numbers under a selected strain amplitude. The pronounced cyclic hardening in the early stage of fatigue resulted in the hysteresis loop at 1% of fatigue life being noticeably higher than the others. Although the hysteresis loops at different cycle numbers varied due to phenomena such as cyclic hardening/softening and elastic modulus degradation under cyclic loading, it can be seen from Figure S3 that the differences between the hysteresis loops from 5% to 90% of fatigue life were not significant. Considering the area of the hysteresis loop, the strain energy density at 1% of fatigue life was 6.15 MJ/m^3^, while the strain energy densities at the other cycle numbers shown in the Figure S3 were all approximately 5.9 MJ/m^3^. This indicates that the hysteresis loop at half-life can represent the stable cyclic state of the sample. Therefore, the hysteresis loop at half-life was used in this study to determine the cyclic strain and stress parameters. Figure 8 shows the hysteresis loops at half fatigue life for 310S and 1045 steels, which were used for the subsequent life assessment. The variation of stress amplitude with loading cycles in the strain-controlled fatigue experiments at selected strain amplitudes for 310S and 1045 specimens is shown in Figure 9. The results suggest that all 1045 specimens displayed overall cyclic softening–hardening behavior, with the degree of cyclic hardening/softening depending on the loading amplitude. The 310S stainless steel exhibited typical cyclic hardening and softening behavior. However, cyclic hardening reemerged when strain amplitudes exceeded 0.7%. The second cyclic hardening observed during the strain-controlled fatigue experiments on stainless steels was primarily associated with cyclic deformation-induced Martensitic transformation. However, according to the EBSD results, the post-test 310S specimen contained less than 1.0% Martensitic phase, indicating that other factors may have contributed to the secondary hardening.

### 3.2. Fracture Morpholog

Fracture surface analysis is an essential method for gaining an in-depth understanding of a material’s fatigue behavior during repeated loading and unloading. It can identify the initiation site of fatigue cracks, locate stress concentration points, or defects, and reveal the direction and rate of crack propagation as well as the microscopic mechanisms of the final fracture. By systematically analyzing the microscopic features of the fracture surface, it is possible to clearly distinguish the crack initiation zone, crack propagation zone, and instantaneous fracture zone, corresponding to different stages of the fracture mechanism. This is of great significance for accurately predicting the fatigue life of materials and optimizing fatigue design.

**Figure 10:**
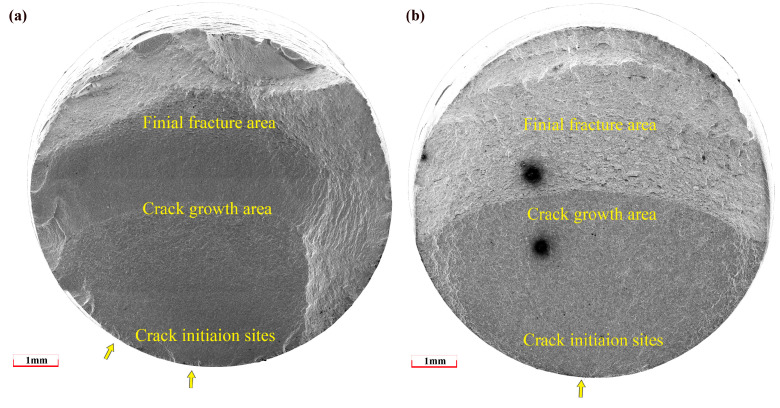
SEM images of fracture profile: (**a**) 310S specimen at Δε/2 = 0.7%, (**b**) 1045 specimen at Δε/2 = 0.5%.

**Figure 11:**
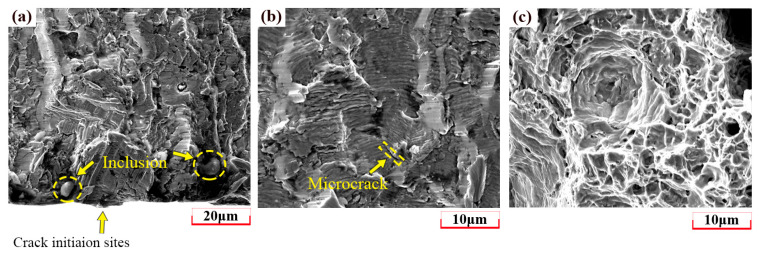
SEM images of 310S specimen at Δε/2 = 0.7%: (**a**) Crack initiation zone, (**b**) crack growth zone, (**c**) final fracture zone.

For both 1045 and 310S specimens, regardless of the strain amplitude, cracks always initiated on the surface of the specimen and then propagated inward. Representative fracture surfaces of these materials are shown in Figure 10 and Figure 11. The fracture surfaces exhibited three regions: the crack initiation zone, the crack propagation zone, and the final fracture zone. The specific locations of crack initiation are indicated by yellow arrows.

Figure 11 shows the magnified SEM images of different fracture surface regions of the 310S specimen at Δε/2 = 0.7%. It is worth noting that there was no significant difference in crack propagation behavior under different strain amplitudes. Obvious intrusion–extrusion marks and inclusions can be observed near the crack initiation zone. The crack propagates in the direction perpendicular to the fatigue striation, and small secondary cracks can also be observed. Figure 11b,c shows the dimples in a typical ductile metallic material.

**Figure 12:**
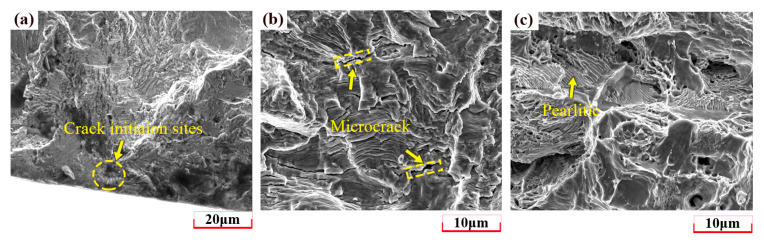
SEM images of 1045 specimen at Δε/2 = 0.5%: (**a**) Crack initiation zone, (**b**) crack growth zone, (**c**) final fracture zone.

Figure 12 shows the magnified SEM images of different fracture surface regions of the 1045 specimen at Δε/2 = 0.5%. The crack initiates from a defect approximately 15 μm below the surface and then propagates inward. Compared with the 310S stainless steel, the 1045 carbon steel clearly exhibited more secondary cracks and more tortuous fatigue striations. In addition, the final fracture region showed not only typical dimples but also pearlite torn apart due to rapid fracturing.

## 4. Fatigue Life Estimation Results

**Figure A1:**
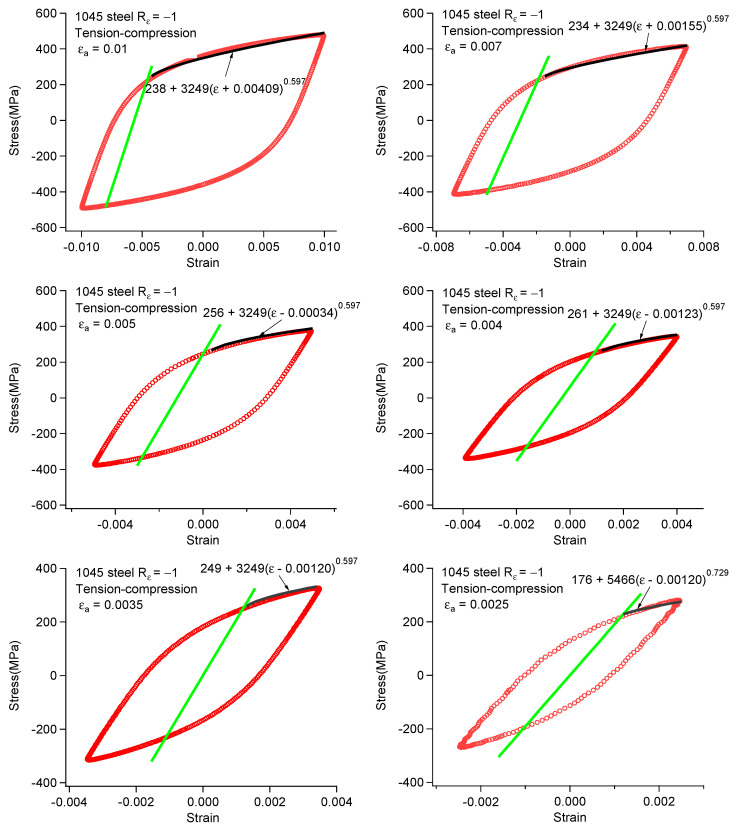

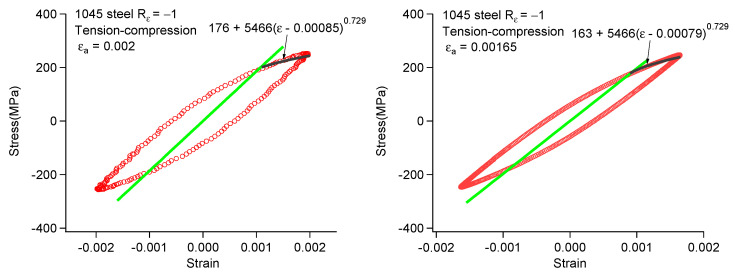
Determination of loop properties for 1045 steel.

**Figure A2:**
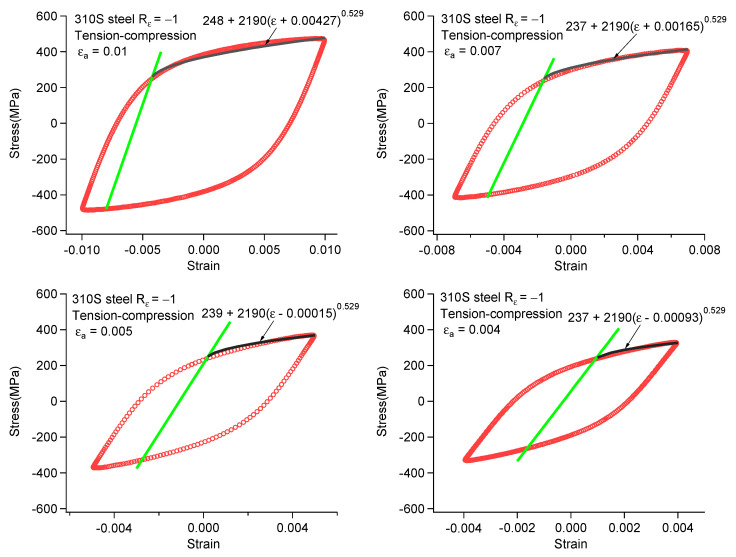

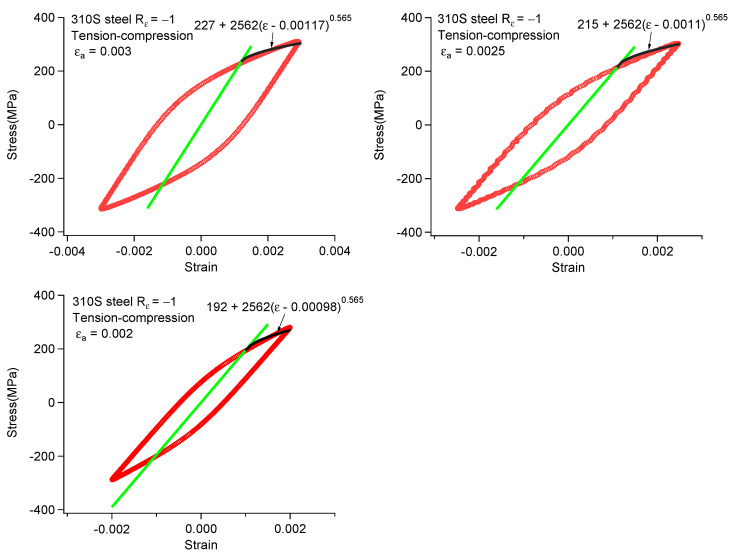
Determination of loop properties for 310S steel.

As shown in Figure 9, the half-life hysteresis loops of 1045 carbon steel and 310S austenitic stainless steel under different strain amplitudes did not exhibit a distinct cyclic yield point. To determine the cyclic yield strain, a 0.2% strain offset line was introduced. However, as described earlier, the 0.2% offset line was no longer applicable when the strain amplitude was relatively low. For 1045 steel, the 0.2% offset line was used when the strain amplitude exceeded 0.35%. When the strain amplitude is below 0.35%, the classical $\varepsilon_{anornimal}^{p}$ should be used. For 310S, the dividing point is a strain amplitude of 0.4%. When the strain amplitude exceeded 0.4%, the 0.2% offset line method was used. When the strain amplitude was below 0.4%, the classical plastic strain amplitude approach was applied. The half-life hysteresis loops of 1045 steel and 310S steel under different strain amplitudes are shown in the Figure A1 and Figure A2. Solid black lines in the Appendix A figures represent the fitting results of Equation (1). The cyclic yield stress $\sigma_{Ycyc}$ and cyclic yield strain $\varepsilon_{Ycyc}$ obtained from the half-life hysteresis loops under different strain amplitudes are summarized in Table 4 and Table 5. In addition, $\sigma_{a}$ in the tables indicates the maximum stress under different strain amplitudes.

**Figure 13:**
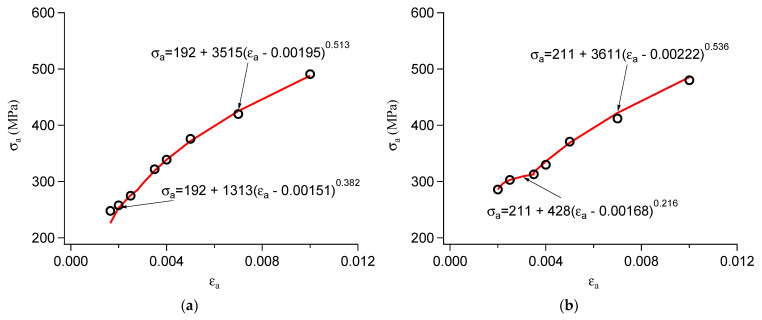
Maximum stress–strain points under different strain amplitudes: (**a**) 1045 and (**b**) 310S steel.

As shown in Equation (2), the cyclic stress–strain relationship can also be described by the maximum stress–strain points of the hysteresis loops under different strain amplitudes. Figure 13 displays the maximum stress–strain points of 1045 carbon steel and 310S stainless steel under different strain amplitudes. Due to the cyclic hardening of 1045 carbon steel, the maximum stress obtained from the half-life hysteresis loops is relatively high at low strain amplitudes. Therefore, two sets of parameters are required to fit Equation (2), as shown in Figure 13a. Similarly, since 310S exhibits secondary hardening at high strain amplitudes and continuous cyclic softening at low strain amplitudes, two sets of parameters are also required to fit Equation (2), as shown in Figure 13b.

Based on the fitting results of Equations (1) and (2), the relationship between $\sigma_{Ycyc}$ and $\varepsilon_{a}$ in Equation (3) was obtained as follows for 1045 carbon steel:

$$\sigma_{Ycyc}=\left\{\begin{array}{ll} 192+3513{\left(\varepsilon_{a}-0.00195\right)}^{0.513}-4762{\left(\varepsilon_{a}-0.00231\right)}^{0.597} & \varepsilon_{a}>0.0035\\ 192+1313{\left(\varepsilon_{a}-0.00151\right)}^{0.382}-8632{\left(\varepsilon_{a}-0.00253\right)}^{0.729} & \varepsilon_{a}\leq 0.0035 \end{array}\right.$$

and as follows for 310S stainless steel:

$$\sigma_{Ycyc}=\left\{\begin{array}{ll} 211+3611{\left(\varepsilon_{a}-0.00222\right)}^{0.536}-3655{\left(\varepsilon_{a}-0.00238\right)}^{0.529} & \varepsilon_{a}\geq 0.004\\ 211+428{\left(\varepsilon_{a}-0.00168\right)}^{0.216}-2293{\left(\varepsilon_{a}-0.000772\right)}^{0.565} & \varepsilon_{a}<0.004 \end{array}\right.$$

**Figure 14:**
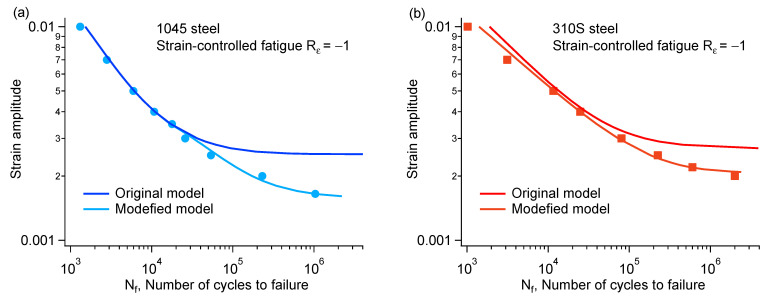
Strain–life prediction curve of (**a**) 1045 and (**b**) 310S steel.

By substituting Equation (3) into Equation (6) for further calculation, the two sets of parameters in Equation (6) for 1045 steel were determined as follows: *β* = 1.25, *γ* = 7.35, *C* = −2.14 × 10^10^ and *β* = −9.35, *γ* = 3.04 × 10^10^, *C* = 1.01 × 10^−14^. The two sets of parameters in Equation (6) for 310S steel were as follows: *β* = 1.25, *γ* = 7.35, *C* = −2.14 × 10^10^ and *β* = −9.35, *γ* = 3.04 × 10^10^, *C* = 1.01 × 10^−14^. The strain–life prediction curves of 1045 and 310S steel are shown in Figure 14a and Figure 14b, respectively. The figures also show the results obtained using the original model. Since the initial model cannot account for the plastic strain at low strain amplitudes, there is a large deviation between the predicted and experimental fatigue lives.

Table 6 presents the relative errors between the experimental fatigue life results and the predictions of the modified model.

**Figure 15:**
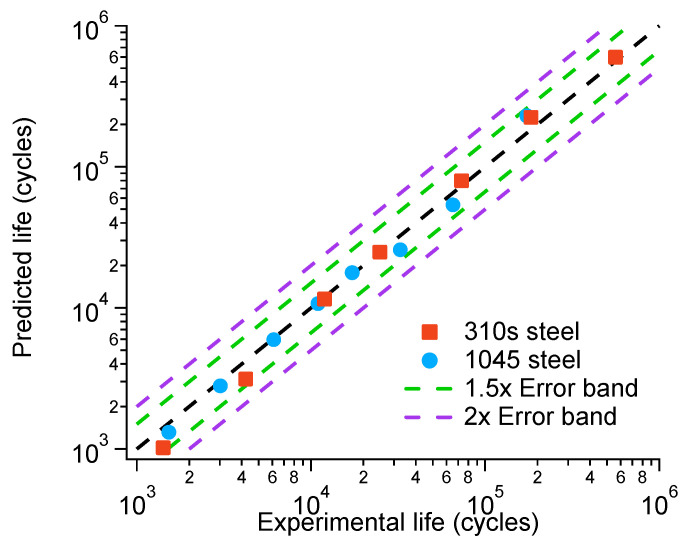
Fatigue life error bands of 310S and 1045 steel.

Using the modified model, the fatigue life at low strain amplitudes can be predicted more accurately, thereby expanding the applicability of the original model. The errors of the modified model are shown in Figure 15. The results showed that all the prediction results fell within the 2-fold error band, and most of them were within the 1.5× error band, indicating that the modified model exhibited good predictive performance.

## 5. Conclusions

In this paper, prediction of the strain–fatigue life of 1045 carbon steel and 310S stainless steel was conducted using a modified model based on the plastic strain and flow stress. The following observations and conclusions were made.

(1) Based on a life prediction model using flow stress, this paper corrects the equation between cyclic plastic strain and flow stress by optimizing the calculation of cyclic plastic strain under different strain amplitudes.
(2) Fatigue test results showed that 1045 carbon steel reached a fatigue life of 10^6^ cycles at a strain amplitude of approximately 0.165%, while 310S stainless steel reached a fatigue life of 10^6^ cycles at a strain amplitude of approximately 0.21%.
(3) The revised model can effectively predict both high-cycle and low-cycle fatigue life across the entire ε-N curve. Compared with the original model, the prediction accuracy for high-cycle fatigue is improved. The error of the revised model is controlled within a 1.5× error band.

## Figures and Tables

**Table 1:** Chemical compositions of 310S stainless steel and 1045 carbon steel (wt.%).

Chemical Compositions	C	Si	Mn	P	S	Cr	Ni	Fe
310S	0.04	0.61	1.06	0.017	0.001	25.65	19.41	balance
1045	0.46	0.27	0.70	0.019	0.027	/	/	balance

**Table 2:** Fatigue results of 310S specimens.

Spec. ID	∆ε/2 (%)	*f* (Hz)	*N_f_* (Cycles)	∆σ/2 (MPa)
Z-04	1.00	0.2	1021	480.0
Z-05	0.70	0.35	3137	412.5
Z-03	0.50	0.6	11,590	370.9
Z-07	0.40	1.0	24,887	330.1
Z-06	0.30	1.0	80,466	312.9
Z-11	0.25	2.0	225,073	308.1
Z-13	0.20	5.0	>2,000,000	285.8

**Table 3:** Fatigue results of 1045 specimens.

Spec. ID	∆ε/2 (%)	*f* (Hz)	*N_f_* (Cycles)	∆σ/2 (MPa)
C-02	1.00	0.2	1316	485.8
C-05	0.70	0.35	2809	414.4
C-03	0.50	0.6	5956	365.5
C-11	0.40	1.0	10,736	344.3
C-12	0.35	1.0	17,820	321.8
C-07	0.25	4.0	53,750	275.3
C-01	0.20	5.0	230,670	257.6
C-08	0.165	5.0	1,049,928	247.8

**Table 4:** Hysteresis properties of 1045 steel.

$\varepsilon_{a}$	0.01	0.007	0.005	0.004	0.0035	0.0025	0.002	0.00165
$\sigma_{Ycyc}$	238	235	256	261	249	208	176	163
$\varepsilon_{Ycyc}$	0.00409	0.00155	−0.00034	−0.00123	−0.001195	−0.001	−0.00085	−0.00079
$\sigma_{a}$	491	410	376	339	322	275	258	248

**Table 5:** Hysteresis properties of 310S steel.

$\varepsilon_{a}$	0.01	0.007	0.005	0.004	0.003	0.0025	0.002
$\sigma_{Ycyc}$	248	237	239	237	227	215	192
$\varepsilon_{Ycyc}$	0.00427	0.00165	−0.00015	−0.00093	−0.00117	−0.0011	−0.00098
$\sigma_{a}$	480	412	371	330	313	303	286

**Table 6:** Relative errors between prediction and experimental results.

Strain Amplitude (%)	Absolute Deviation Value (%)	
	1045	310S
1.0	15.88	310S
0.7	7.16	38.39
0.5	2.51	34.17
0.4	2.06	3.01
0.35	4.62	3.49
0.3	/	/
0.25	21.23	8.68
0.2	24.93	18.68
0.165	2.99	19.5

## Data Availability

The original contributions presented in this study are included in the article/Supplementary Materials. Further inquiries can be directed to the corresponding author.

## Supplementary Materials

The following supporting information can be downloaded at https://www.mdpi.com/article/10.3390/ma18143274/s1, Figure S1. Cross-sectional SEM image of the 1045 specimen. Figure S2. KAM image of (**a**) 1045 and (**b**) 310S specimens before fatigue test. Figure S3. Hysteresis loops of 310S at different cycle numbers under a strain amplitude of 0.7%.

## Appendix A

The half-life hysteresis loop of 1045 and 310S steel under different strain amplitudes is shown in Figure A1 and Figure A2. The red color in the figure represents the hysteresis loop, while the green color indicates the offset line used to determine the cyclic plastic strain and stress.
