# Supplementary material for: A Modified Fatigue Life Prediction Model for Cyclic Hardening/Softening Steel
Source: Materials (Basel). 2025 Jul 11;18(14):3274. doi: 10.3390/ma18143274 (PMC12298996; doi:10.3390/ma18143274)
Supplement: Supplementary file 1 [file materials-18-03274-s001.zip › materials-3665519-supplementary.pdf]

materials

Supplementary Materials

The etched cross-sectional 1045 specimens were observed under SEM. Figure S1 shows a microstructure consisting of ferrite and layered pearlite structures of 1045 steel.

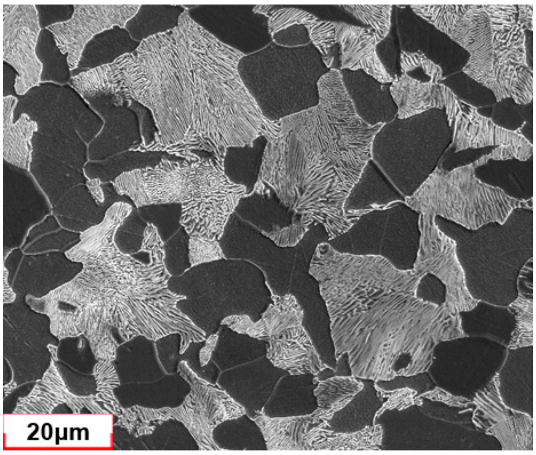

Figure S1. Cross-sectional SEM image of the 1045 specimen.

Figure S2 shows the KAM image of 1045 and 310S specimens before fatigue test. The dislocation density in the initial specimens is very low.

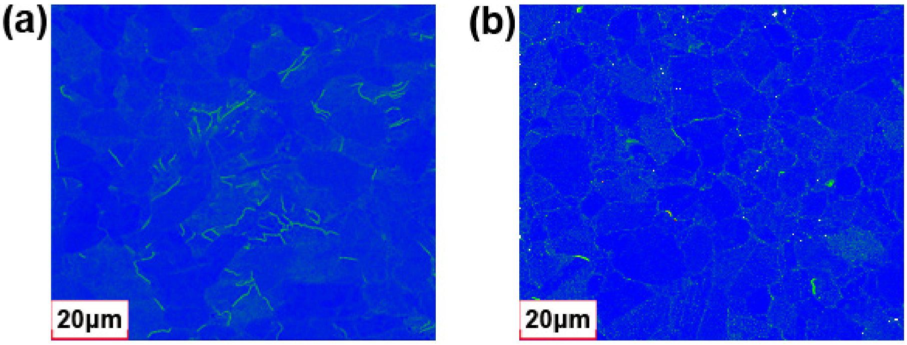

Figure S2. KAM image of (a)1045 and (b)310S specimens before fatigue test.

Figure S3 shows the hysteresis loops of 310S specimens at different cycle numbers under a selected strain amplitude.

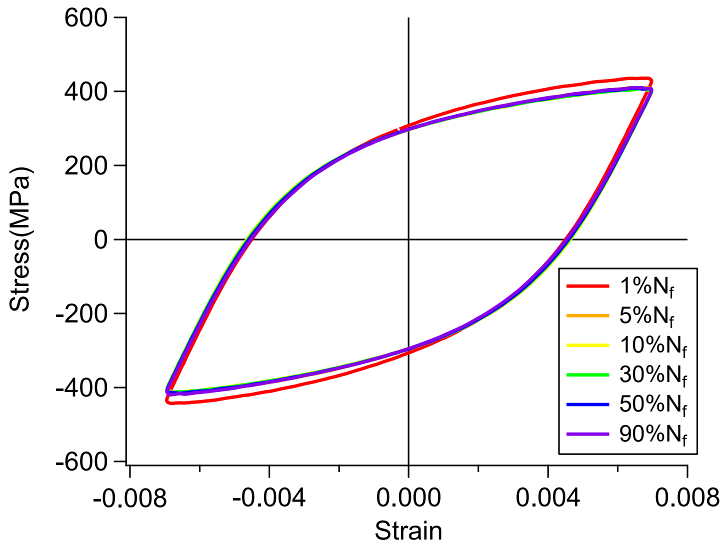

Figure S3. Hysteresis loops of 310S at different cycle numbers under a strain amplitude of 0.7%.
